# Stress, anxiety and depression in parents of children with autism spectrum disorders in Kazakhstan: prevalence and associated factors

**DOI:** 10.1017/gmh.2022.51

**Published:** 2022-10-11

**Authors:** Raushan Alibekova, Chee Kai Chan, Byron Crape, Kainar Kadyrzhanuly, Arnur Gusmanov, Sofiya An, Sholpan Bulekbayeva, Zulfiya Akhmetzhanova, Assel Ainabekova, Zhanibek Yerubayev, Fariza Yessimkulova, Aislu Bekisheva, Zarina Ospanova, Makhabbat Rakhimova

**Affiliations:** 1 Department of Medicine, School of Medicine, Nazarbayev University, Astana, Kazakhstan; 2 Department of Biomedical Sciences, School of Medicine, Nazarbayev University, Astana, Kazakhstan; 3 School of Humanities and Social Sciences, Nazarbayev University, Astana, Kazakhstan; 4 National Children's Rehabilitation Center, Corporate Fund “University Medical Center”, Astana, Kazakhstan; 5 Master of Public Health Program, School of Medicine, Nazarbayev University, Astana, Kazakhstan

**Keywords:** Anxiety, autism spectrum disorder, caregivers, depression, stress

## Abstract

**Introduction:**

Studies worldwide reported increased levels of stress among parents of children with autism due to the unique caregiving challenges. While research has shown that parents' and autistic child's demographics and behavioral characteristics are associated with psychological distress among caregivers of children with autism, very few studies have investigated the impact of the caregiver's unmet needs on various aspects of the perceived family burden.

**Methods:**

This cross-sectional study examined the prevalence of stress, anxiety and depressive symptoms among a wide range of 146 parents with different sociodemographic characteristics, social support and unmet needs who care for children with autism spectrum disorder. These parents were recruited from autism non-governmental organizations and the National Children's Rehabilitation Center in Astana, Kazakhstan, a post-Soviet country in Central Asia. Multiple linear regression analyses were utilized to examine the relationship of parental psychological distress variables with social support, unmet needs and parental and child sociodemographic characteristics.

**Results:**

Significantly higher levels of stress and depression were reported among parents who perceived their needs as being unmet or extremely unmet as addressed by societal acceptance as compared to parents who reported adequate levels of needs met by social acceptance. Employed parents and parents with a higher level of perceived friends' support had less symptoms of stress, anxiety, and depression.

**Conclusions:**

Increasing public awareness about autism and providing early detection and interventions for distressed caregivers of children with autism may be helpful in improving healthy functioning of parents and the entire family.

## Introduction

Caregiving of children diagnosed with autism spectrum disorder (ASD) is challenging due to the heterogeneous and chronic nature of autism, and broad range of possible co-occurring conditions (Vohra *et al*., 2014). Lack of the child's communication and socialization skills, low ability for self-caring in daily routines, and societal barriers such as inadequate school system services to address needs and lack of other support resources may challenge families raising a child with ASD (Ho *et al*., 2018). Due to the multifaceted nature of the disorder and lack of evidence-based treatment options, parents of autistic children are in a persistent search for services from multiple sources and specialists to address the needs of their children (An *et al*., 2020; Srinivasan *et al*., 2021).

Population-based studies from the US showed that despite greater utilization of services, caregivers of children with ASD were more likely to report difficulties in accessing child healthcare services and have more unmet family support needs as compared to caregivers of children with other developmental disabilities or typically developing children (Vohra *et al*., 2014; Srinivasan *et al*., 2021). Previous research from low and middle-income countries on children with ASD found challenges related to primary caregivers' needs for family support, accessibility for services and well-trained specialists, public awareness and understanding of autism, inclusive education system and social acceptance of children with ASD (Bilgin and Kucuk, 2010; Divan *et al*., 2012; Tilahun *et al*., 2016; Gobrial, 2018; Shorey *et al*., 2020). Emotional burden on parents due to the child's disorder and parental mental health were identified as unmet needs by studies conducted in both high and lower resourced settings (Divan *et al*., 2012; Lushin and O'Brien, 2016; Tilahun *et al*., 2016; Papadopoulos, 2021).

Extensive research has found that parents of children with ASD often experience elevated levels of stress, depression and anxiety, as compared to parents of typically developing children (Hodge *et al*., 2011; Bitsika *et al*., 2013; Wang *et al*., 2013; Lai *et al*., 2015; Al-Farsi *et al*., 2016; Padden and James, 2017). Though the majority of studies investigating parental stress due to having a child with ASD were conducted in Western or European contexts (Hayes and Watson, 2013; Barroso *et al*., 2018), similar findings from systematic reviews of studies in Southeast Asia (Ilias *et al*., 2018) and Arab countries (Alkhateeb *et al*., 2022) also show increased stress levels for parents and other adverse impacts on their mental health.

Kazakhstan is a post-Soviet upper-middle income country located in Central Asia (World Bank, 2022). With a total population of 19 million, children aged from 0 to 17 years constitute about 32% of Kazakhstani population (Bureau of National Statistics Agency for Strategic Planning and Reform, Republic of Kazakhstan, 2022). Officially the estimated prevalence of ASD is 2.6 per 100 000 children in Kazakhstan (Perfilyeva *et al*., 2019), which is lower than prevalence found in most countries worldwide. The low rates of diagnosis have been linked to gaps in medical training and lack of expertise among professionals (Somerton *et al*., 2022) and low public awareness and social stigma attached to ASD in Kazakhstan (An *et al*., 2018, 2020). Public services for children with autism in Kazakhstan are in the early stages of gradual development. During the last two decades, governmental regulations were introduced to increase detection, treatment, and provision of social services for children with special needs (An *et al*., 2018). Recently, the government of Kazakhstan introduced a road map for advancing healthcare provisions for ASD children for 2019–2020, committing to improve the quality of life of both children with autism and their families through early diagnosis, rehabilitation, inclusive education and social integration (An *et al*., 2020). Educational and recreational facilities, and community-based non-profit organizations have shaped most of the infrastructure for care provisions for autistic children (An *et al*., 2018; Somerton *et al*., 2022). In recent years a substantial upsurge in the number of registered children with autism has been observed, increasing from 77 in 2003 to approximately 2000 in 2007 (An *et al*., 2018; National Centre for Correctional Pedagogy), and to nearly 4887 in 2021 (Republican Scientific and Practical Center for Mental Health, reported by national media). Despite this apparent upsurge in numbers, there has been a sparsity of research in autism in Kazakhstan. To the authors' knowledge, limited qualitative and mixed-methods studies have explored the experiences of parents raising a child with ASD (An *et al*., 2018, 2020) and knowledge and beliefs of health specialists involved in diagnosis of children with autism (Somerton *et al*., 2022). The qualitative study by An *et al*. (2018), investigating parental perspectives on support and services for children with autism in Kazakhstan, found challenges and unmet needs related to early diagnosis, and availability of trained specialists, financial support, multidisciplinary team of specialists, inclusive education, and acceptance of autistic children by society. However, the mental health of parents with autistic children was not directly addressed.

Increased caregiver stress or depression has been shown to decrease parenting abilities (Cummings and Davis, 1994), which can in turn negatively impact the child's physical and mental health (Olfson *et al*., 2003; Lushin and O'Brien, 2016). Researchers found that parenting stress was inversely associated with successful outcomes of early learning processes of autistic children (Osborne *et al*., 2008) and was strongly directly associated with the development of depressive symptoms and deterioration of the quality of life in parents of children with high-functioning pervasive developmental disorders (Suzumura, 2015). A large-scale cohort study conducted by Fairthorne *et al*. (2014) showed that caregivers of children with ASD had higher risk of death and experienced substantially poorer survivorship.

Factors related to the child with ASD and parents, and social level factors were found to be predictors of the psychological burden for caregivers of children with ASD. Increased severity of autism symptoms (Ekas and Whitman, 2010; Picardi *et al*., 2018), number of co-occurring conditions (Zablotsky *et al*., 2013) and children's emotional and behavioral problems (Estes *et al*., 2013; Falk *et al*., 2014; Zhou *et al*., 2019) were found to be predictors of stress, anxiety and depression among caregivers of children with ASD. Parental perceived limit-setting ability, satisfaction with parenting, mothers' age (Falk *et al*., 2014), self-stigma (Werner and Shulman, 2013; Chan and Lam, 2018) and poor coping strategies (Whitehead *et al*., 2015) were also found to be associated with depression among parents. Father's age (Falk *et al*., 2014) and financial strain were predictors of higher parental stress levels (Zablotsky *et al*., 2013; Falk *et al*., 2014; Ilias *et al*., 2018). Poor quality interactions within the extended family and absence of children's schooling were also associated with higher parental stress (Derguy *et al*., 2016). Public stigma due to autism was associated with depression and anxiety (Gray, 2002; Chan and Lam, 2017).

Some studies found that not all caregivers of children with ASD experience increased stress levels and elevated depression and anxiety symptoms. Some families display traits of resilience, such as family hardiness, which was inversely associated with depression (Weiss, 2002). Perceived social support (Ekas *et al*., 2010; Weiss *et al*., 2013; Zablotsky *et al*., 2013; Whitehead *et al*., 2015; Drogomyretska *et al*., 2020), psychological resilience (Bitsika *et al*., 2013), religious beliefs (Ilias *et al*., 2018), effective coping skills (Zablotsky *et al*., 2013) and participation in parental support groups (Kerr and McIntosh, 2000) may play a role in protecting parental mental health and wellbeing of caregivers of autistic children.

While a considerable amount of research investigates risk factors for psychological distress among caregivers of children with ASD, the focus of research on the experiences of parents in the upper-middle income Central Asian country of Kazakhstan is lacking. Furthermore, though a large part of previous research has globally shown that parent and autistic child demographics and behavioral characteristics are associated with psychological distress among caregivers of children with autism, a sparsity of studies have investigated the impact of caregivers' unmet needs on psychological burden. Our study addresses this gap by introducing a more comprehensive list of potential risk and protective factors for the evaluation of their associations with psychological distress.

The aims of our study were to (1) determine the prevalence of stress, anxiety, and depressive symptoms among parents of children with ASD, and to (2) examine the associations of a more comprehensive range of potential risk and protective factors associated with these outcomes, including sociodemographic characteristics of parents and autistic children, unmet needs of social support and acceptance by society, financial support, and use of various treatment and behavioral interventions. Findings of our study will provide insights into factors associated with psychological distress in parents of autistic children in the Central Asian region, an under-researched area. Findings of this study can provide a foundation to raise public awareness of the unmet psychological needs of parents raising children with ASD, and provide evidence to inform the planning of more effective policy and family focused interventions.

## Methods

### Study setting and sample

Our study participants consisted of 146 parents of autistic children. Recruitment of study participants to this cross-sectional survey was conducted by convenience sampling from various places in Astana city, the capital of Kazakhstan, at the awareness-raising event on the International Autism Day, the Public Association of Parents of Children with Autism ‘ORDA’, and the National Children's Rehabilitation Center, which provides services for children with ASD countrywide. Data collection was conducted during the months April to December 2018. Participants met the following inclusion criteria: parents having at least one autistic child and could communicate in Russian or Kazakh language(s). All participants were asked for an oral consent and were not asked to sign a written informed consent form, due a reluctance to sign forms as a result of the legacy of the Soviet Union. An information letter as part of the oral consent process was provided to communicate objectives, risks and benefits with participation in this study, as well as covering the rights of participants and responsibilities of the researcher. After providing oral informed consent, parents completed an anonymous paper-based survey. This survey was administered to participants who provided consent at convenient private spaces at the ‘ORDA’ Center and at the National Children's Rehabilitation Center. The study was approved by the Ethical Committee of the Corporate Fund ‘University Medical Center’ (IRB №: 6/04092018).

### Instruments

The parent self-administered survey comprised of 59 questions inquiring about the sociodemographic characteristics of parents and their autistic children, parental needs with regards to autism care, membership in parental support groups, and two standardized scales to assess parental mental health and perceived social support.

The survey was administered in two languages, Kazakh and Russian. The original English version of the standardized scales were translated into Kazakh language by our multilingual research team members, and were culturally adapted to ensure validity and reliability of the tool, following the guidelines for cross-cultural adaptation of self-report measures (Beaton *et al*., 2000). We used a Russian version of the standardized scales that was validated by previous researchers. The survey was pretested and corrected for clarity and understanding among a small sample of parents of autistic children; pre-test surveys were excluded from analysis.

### Depression anxiety stress scale (DASS-21)

The DASS-21 is an internationally-validated tool used to assess the mental health of an individual based on negative emotional states of depression, anxiety and stress experience over the past week (Lovibond and Lovibond, 1995). The DASS is 21-item questionnaire divided into three sub-scales, each containing seven questions. The Depression subscale assesses hopelessness, devaluation of life, self-abasement, lack of interest, and inertia. The Anxiety subscale evaluates panic attacks, skeletal muscle effects, and subjective experience of anxious affect. The Stress subscale assesses levels of chronic difficulty in relaxing, nervous arousal, and being easily upset, irritable and impatient. Each question is measured on a four-point Likert scale ranging from 0 (‘did not apply to me at all’) to 3 (‘applied to me very much’). Overall score is calculated by summing the scores of each emotional state sub-scale, then categorized into none, mild, moderate, severe-and-extremely severe symptoms of depression, anxiety, or stress. The DASS-21 showed good internal reliability in previous studies, with Cronbach's *α* of 0.835, 0.737 and 0.761 for depression, anxiety and stress subscales in one study (Le *et al*., 2017). For the current study, we translated and culturally adapted the scale in the Kazakh language. We also utilized the Russian version of the DASS-21, which was validated by the National Centre in HIV Epidemiology and Clinical Research in Sydney Australia as part of an international trial (Psychology Foundation of Australia, 2018). In our sample, Cronbach's *α* for the overall scale was 0.936 and 0.942 for Kazakh and Russian versions, respectively.

### Multidimensional Scale of Perceived Social Support

The Multidimensional Scale of Perception of Social Support was developed by Zimet *et al*., in 1988, with the Russian language version previously being tested in a study by Kuznetsov *et al*. (2015). The Scale is a short self-report measure that assesses subjective perceptions of social support, which is composed of 12 items rated on a 7-point Likert scale, ranging from 1 for ‘absolutely disagree’ to 7 for ‘absolutely agree’. Based on the pre-test of the questionnaire, for the current study the response scale was reduced up to 5 points to improve the understanding and acceptability of the scale. Thus, the summed total score ranges from 12 to 60, where the higher score indicates more social support. The scale assesses effectiveness and adequacy of social support in three dimensions - ‘family’, ‘friends’, and ‘significant other’. The scale is considered by Zimet *et al*. (1988) as a psychometric probe with good reliability (Cronbach's *α* = 0.88), adequate factor and construct validity, and simple for examination and a time-saving tool. In our sample Cronbach's *α* was 0.926 and 0.954 for Kazakh and Russian language versions, respectively.

The sociodemographic section of the questionnaire collected information about parental age, gender, education, marital status, number of children, family income, employment status, place of residence, and financial and instrumental care support (10 questions). This section also included questions concerning the autistic child's age, gender, and diagnosis, the specialists who informed parents about ASD for the first time, living arrangements for the child, co-occurring chronic conditions, educational attainment (home-based/caregiver, mixture of classes, regular education classroom, and special educational classroom), and use of various treatment interventions (eight questions).

A question on treatment interventions received in the past year included Applied Behavior Analysis (ABA) therapy, academic classes (sessions with specialists, such as speech therapist and psychologist), occupational therapy, physical therapy (e.g. usage of light, heat, ultrasound, electrical impulses) and social skills interventions. Parents were asked if they participated in parental support organizations (one item). A few open-ended explorative questions about perceived benefits and barriers for participation in support groups were asked additionally.

Parents' perceived needs fulfillment was assessed by seven items, inquiring about early diagnosis, availability of trained specialists, financial support, inclusive education, acceptance by society, support of parental organizations, and multidisciplinary team of specialists. The research team created this measure based on findings of the previous qualitative study by An *et al*., 2018, which identified the most salient unmet needs of parents of autistic children in Kazakhstan. The extent of perceived fulfillment of needs was measured using following scale: ‘fully met’ = 1, ‘partially met’ = 2, ‘unmet’ = 3 and ‘extremely unmet’ = 4. Each item on this scale was analyzed as a separate categorical variable.

### Data analysis

Descriptive statistics were calculated to characterize the study sample using percentages for categorical variables, and means and standard deviations for continuous variables. χ^2^ analysis was performed to test statistical significance of categorical variables, with Fisher's exact test (two-tailed) substituted when assumption of expected small cell size was not met. The differences for continuous independent variables between groups were assessed by the Student's *t* test, with ANOVA utilized when more than two groups were to be compared. Multivariate linear regression analyses were performed to explore the associations of parental mental health status with independent variables, where three separate models were constructed for depression, anxiety, and stress. Due to limited sample size the *p* value of 0.25 in bivariate analysis was selected for inclusion of variables into final models, after which backward stepwise selection was performed to determine models. Mean differences and 95% confidence intervals were chosen as measures of association between independent and outcome variables. Statistical significance was set at *α* = 0.05. All statistical analyses were completed using STATA Software version 15.1.

## Results

### Socio-demographic and children's illness-related characteristics

Frequency distributions of socio-demographic characteristics of parents and their children with autism, as well as children's illness-related characteristics, are presented in Table 1.

**Table 1.** Descriptive characteristics of parents and children with autism spectrum disorder (ASD) (*n* = 146)

Characteristics of parents	*n*^a^ (%)
Age, Mean ± s.d. (years)	35.7 ± 6.51
Gender	
Male	14 (10.1%)
Female	124 (89.9%)
Highest completed degree	
High School	55 (39%)
University	86 (60%)
Employment	
No	95 (67.9%)
Yes	45 (32.1%)
Marital status	
Married	124 (88.6%)
Single	16 (11.4%)
Number of children	
⩽2	97 (66.44%)
>2	49 (33.56)
Family monthly income (tenge)	
< 150 000	85 (62.5%)
⩾150 000	51 (37.5%)
Additional financial coverage	
Any coverage	122 (89.7%)
None	14 (10.3%)
Place of residence	
Astana city	88 (64.2%)
Other regions	49 (35.8%)
Person helps in taking care	
Having hired person	15 (11.5%)
Not having hired person	115 (88.5%)
Member of parental support group	
No	76 (54.7%)
Yes	63 (45.3%)
Barriers to become a member of support group	
No barriers	10 (8.7%)
Having barriers	105 (91.3%)

Characteristics of children	*n* (%)
Age, Mean ± s.d. (years)	7.11 ± 2.83
Gender	
Male	108 (75.5%)
Female	35 (24.5%)
Placement at home	
Yes	140 (97.9%)
No	3 (2.1%)
Diagnosis	
Autistic disorder	136 (95.8%)
Asperger syndrome	1 (0.7%)
Other pervasive developmental disorders	5 (3.5%)
Informed about ASD for the first time	
Neurologist	57 (40.4%)
Psychiatrist	38 (26.9%)
Psychotherapist	13 (9.2%)
Pediatrician	12 (8.5%)
On their own	8 (5.7%)
Psychologist	7 (4.9%)
Educational professional	6 (4.3%)
Education	
Home-based	36 (33%)
Mixture of classes	13 (11.9%)
Regular education classroom	15 (13.8%)
Special educational classroom	45 (41.3%)
Co-occurring chronic conditions	
No	104 (81.2%)
Yes	24 (18.8%)

a Total counts and percentages may vary because of missing values.

Women constituted a significant proportion of the respondents (89.9%) with overall mean age of 35.69 years (s.d. ± 6.51). Almost nine-out-of-ten respondents were married (88.6%), and over half of these parents held university degrees (60.0%) and were residents of Astana city (64.2%). Around two thirds of them were not employed (67.9%), with monthly family income of less than 150000 tenge (~ 390 USD, 62.5%).

A total of 75.5% of parents reported that they were raising a boy with autism and that the mean age of their children was 7.11 years (s.d. ± 2.83). A total of 95.8% of respondents indicated that their child has Autistic Disorder, with 3.5% indicating other pervasive developmental disorders (3.5%) and 0.7% indicating Asperger Syndrome. Nearly 19% stated that their child had at least one other co-occurring chronic condition such as epilepsy, allergy, impaired or loss of hearing, and congenital malformation. A total of 40.4% of parents were first informed about their child's diagnosis by a neurologist and 26.9% by a psychiatrist. A total of 95.8% of children with ASD were living at home and 33% stayed at home to receive interventions. A total of 11.9% attending a mixture of classes and the remaining children attended special or regular educational classrooms, 41.3% and 13.8%, respectively.

### Membership in support groups, received interventions and needs of parents

A total of 45.3% of respondents were members of support groups for parents with autistic children (Table 1). Out of all respondents, 73.3% indicated that barriers exist that hinder becoming a member of parental support groups, such as lack of information on the support groups and travel distance to support group meetings.

Frequency distributions of treatments and behavioral interventions are provided in Table 4. A majority of children with autistic disorder were engaged in academic classes (82.9%), 39.7% received occupational therapy and 36.3% received social skills interventions. About one quarter (26.0%) of the respondents' children had access to ABA and the same percentage (26.0%) had access to physical therapy. The need for inclusive education was the most frequently reported unmet-or-extremely-unmet need as compared to other needs, accounting for 42.5% and 24.7% of respondents, respectively (Table 5). Approximately a third of caregivers mentioned early diagnosis (31.5%), financial support (31.5%) and acceptance by society (33.3%) as unaddressed needs. While a substantial percentage of respondents identified early diagnosis and acceptance by society as extremely-unmet needs (8.8% and 11.0%, respectively), unmet financial support need was reported by a higher 19.3% of respondents. In addition, comparatively fewer respondents were unsatisfied by the level of availability of trained specialists (24.03%), support from parental organizations (27.59%), and multidisciplinary team of specialists (20.00%). Just over one-tenth of respondents had hired a person to assist in caring for the child with ASD (11.5%). The majority received additional financial coverage for their children's needs and services (89.71%); in most cases, these were governmental pension and governmental special social service funds.

### Stress, anxiety and depression: prevalence and associations

A total of 136 complete responses were analyzed to calculate scores for outcome variables. Overall, the combined prevalence for mild to extremely severe levels of stress, anxiety and depression were 61, 52.9, and 53.7%, respectively (Table 2).

**Table 2.** Stress, anxiety, and depression among parents of children with ASD

Level of severity	Stress *n*(%)	Anxiety *n*(%)	Depression *n*(%)
None	53 (39%)	64 (47.1%)	63 (46.3%)
Mild	31 (22.8%)	7 (5.1%)	22 (16.2%)
Moderate	26 (19.1%)	26 (19.1%)	25 (18.4%)
Severe	15 (11%)	18 (13.2%)	18 (13.2%)
Extremely severe	11 (8.1%)	21 (15.4%)	8 (5.9%)
**Total**	**136 (100%)**	**136 (100%)**	**136 (100%)**

The results of the bivariate analysis using simple linear regression are presented in Table 3. Parents' age, gender, family income and membership in support groups were not associated with either of DASS's outcomes. Employed parents were less stressed and depressed than parents who were unemployed. Parents from Astana city were more anxious and depressed in comparison with their counterparts from other regions. Also, respondents who had not hired a person to assist in caring for their ASD child were found to be more stressed than those who had hired someone (all *p* values < 0.05). Among children with autism, having co-occurring conditions was associated with a higher level of parental stress.

**Table 3.** Psychological distress among parents in relation to parental and child's characteristics utilizing bivariate simple linear regression analysis

Variables	Stress		Anxiety		Depression	
	*β* (coef.)	*p* value	*β* (coef.)	*p* value	*β* (coef.)	*p* value
Age of participating parent	0.043	0.773	0.091	0.459	0.0086	0.941
Gender of participating parent						
Female	Ref.		Ref.		Ref.	
Male	−5.82	0.079	−5.49	0.054	−3.81	0.159
Employment of participating parent						
No	Ref.		Ref.		Ref.	
Yes	−5.89	0.005	−3.259	0.061	−3.578	0.035
Residence of participating parent						
Other region	Ref.		Ref.		Ref.	
Astana city	2.83	0.178	4.708	0.006	4.804	0.004
Support in taking care						
Having hired person	Ref.		Ref.		Ref.	
Not having hired person	8.77	0.008	1.582	0.556	3.158	0.243
Family monthly income						
< 150 000	Ref.		Ref.		Ref.	
> 150 000	0.986	0.627	1.701	0.314	1.65	0.311
Age of child with autism	−0.32	0.346	−0.175	0.555	−0.065	0.813
Child with autism living at home						
Yes	Ref.		Ref.		Ref.	
No	−10.70	0.107	−5.57	0.311	−7.89	0.139
Child with autism with co-occurring chronic condition						
No	Ref.		Ref.		Ref.	
Yes	5.98	0.026	2.55	0.256	3.597	0.102
Member in parental support group						
Yes	Ref.		Ref.		Ref.	
No	4.77	0.20	3.27	0.298	2.38	0.436

Parents with children receiving ABA therapy were reported to be more likely to have mood disorders than those who did not receive ABA therapy (Table 4).

**Table 4.** Parental psychological distress by treatment and behavioral interventions utilizing bivariate simple linear regression analysis

Treatment or intervention	Stress		Anxiety		Depression	
	*β* (coef.)	*p* value	*β* (coef.)	*p* value	*β* (coef.)	*p* value
ABA therapy (146)						
No (74.0%)	Ref.		Ref.		Ref.	
Yes (26.0%)	6.64	0.003	5.32	0.003	4.08	0.024
Academic classes (146)						
No (17.1%)	Ref.		Ref.		Ref.	
Yes (82.9%)	−2.77	0.298	−2.10	0.337	−2.21	0.301
Occupational therapy (146)						
No (60.3%)	Ref.		Ref.		Ref.	
Yes (39.7%)	−0.13	0.946	−0.27	0.865	0.09	0.953
Physical therapy (146)						
No (74.0%)	Ref.		Ref.		Ref.	
Yes (26.0%)	0.64	0.769	−1.26	0.481	−0.19	0.912
Social skills intervention (146)						
No (63.7%)	Ref.		Ref.		Ref.	
Yes (36.3%)	−3.35	0.097	−2.39	0.149	−2.95	0.068
Physical training (146)						
No (91.8%)	Ref.		Ref.		Ref.	
Yes (8.2%)	−1.75	0.625	−1.99	0.501	−1.93	0.505

Table 5 shows that those respondents who perceived that acceptance by society was unmet-or-extremely-unmet were more depressed, anxious, and stressed than parents who reported that acceptance by society was satisfactory. The more support parents reported receiving from their family, friends, and significant others, the lower the levels of depression, anxiety, and stress they experienced.

**Table 5.** Parental psychological distress by perceived fulfillment of needs utilizing bivariate simple linear regression analysis

Needs (Total = 146)	Stress		Anxiety		Depression	
	*β* (coef.)	*p* value	*β* (coef.)	*p* value	*β* (coef.)	*p* value
Early diagnosis (124)						
Met (59.7%)	Ref.		Ref.		Ref.	
Unmet (31.5%)	0.92	0.673	−2.14	0.239	−1.53	0.386
Extremely unmet (8.8%)	−2.35	0.521	−4.59	0.131	−2.37	0.426
Trained specialists (121)						
Met (69.8%)	Ref.		Ref.		Ref.	
Unmet (24%)	4.18	0.088	2.79	0.163	2.81	0.154
Extremely unmet (6.2%)	1.61	0.723	−0.26	0.945	0.84	0.818
Financial support (105)						
Met (49.2%)	Ref.		Ref.		Ref.	
Unmet (31.5%)	1.82	0.436	0.71	0.715	1.27	0.505
Extremely unmet (19.3%)	−0.05	0.984	−1.66	0.469	−0.17	0.938
Inclusive education (101)						
Met (32.8%)	Ref.		Ref.		Ref.	
Unmet (42.5%)	0.39	0.864	−0.75	0.699	−0.08	0.964
Extremely unmet (24.7%)	1.31	0.624	−2.21	0.322	0.26	0.904
Acceptance by society (112)						
Met (55.7%)	Ref.		Ref.		Ref.	
Unmet (33.3%)	6.53	0.003	4.68	0.012	4.67	0.008
Extremely unmet (11%)	8.15	0.015	5.12	0.074	7.87	0.004
Support from families (134)	−0.50	0.047	−0.51	0.013	−0.54	0.006
Support from friends (131)	−0.65	0.003	−0.64	0.001	−0.53	0.002
Support from significant other (129)	−0.48	0.050	−0.51	0.010	−0.50	0.009
Support from parental support groups (108)						
Met (57.5%)	Ref.		Ref.		Ref.	
Unmet (27.5%)	2.16	0.357	−0.3	0.88	−0.07	0.97
Extremely unmet (15%)	3.86	0.194	1.57	0.535	2.37	0.334
Multidisciplinary team (124)						
Met (71.8%)	Ref.		Ref.		Ref.	
Unmet (20%)	2.69	0.289	3.13	0.134	2.71	0.183
Extremely unmet (8.2%)	0.75	0.843	−2.07	0.507	0.08	0.978
Having barriers to become a member of parental support group (130)						
No (8.7%)	Ref.		Ref.		Ref.	
Yes (91.3%)	4.769	0.200	3.26	0.298	2.384	0.436

### Multivariate linear regression analysis

For each of the DASS's outcomes (depression, anxiety and stress), a separate linear regression model was generated. Three variables, employment of participating parent, support of friends, acceptance by society, were statistically significantly associated with at least one outcome variable (Table 6).

**Table 6.** Factors associated with parental psychological distress utilizing multivariate linear regression analysis

Variables	Stress level Model 1		Anxiety level Model 2		Depression level Model 3	
	Coefficient	*p* value	Coefficient	*p* value	Coefficient	*p* value
Employment						
No	Ref.		Ref.		Ref.	
Yes	−5.58	0.016	−2.06	0.341	−3.04	0.118
Place of residence						
Other regions	Ref.		Ref.		Ref.	
Astana city	−0.56	0.793	2.95	0.144	2.37	0.190
Support of friends	−0.53	0.014	−0.41	0.044	−0.41	0.025
ABA therapy						
No	Ref.		Ref.		Ref.	
Yes	4.09	0.083	4.20	0.059	2.60	0.190
Child with autism with chronic condition						
No	Ref.		Ref.		Ref.	
Yes	3.31	0.230	0.62	0.810	1.54	0.507
Acceptance by society						
Met	Ref.		Ref.		Ref.	
Unmet	5.07	0.024	3.11	0.139	3.43	0.069
Extremely unmet	11.86	0.002	6.15	0.084	9.94	0.002

The strongest association with stress was observed for perceived level of acceptance by society; parents reporting level of acceptance by society as an extremely-unmet need were more stressed than those reporting this need as adequately met (mean difference 11.86, *p* value 0.002). Similarly, there was also a strong association for extremely-unmet need of acceptance by society with depression (mean difference 9.94, *p* value 0.002). Also, employed parents had lower stress levels than unemployed respondents. A higher level of perceived friends' support was found to be associated with lower levels of stress, anxiety and depression (coefficients: −0.53 for stress, −0.41 for anxiety and depression with p-values 0.014, 0.044 and 0.025, respectively). Additional analysis in our study found that children who received ABA therapy were more likely to have chronic conditions; in the final multivariate analysis that including confounders parent's employment status and social support, ABA was no longer associated with parental stress and mood disorders.

## Discussion

To our knowledge this cross-sectional survey, utilizing internationally validated scales, is the first study conducted in a post-Soviet Central Asian country to investigate a wide range of potential risk and protective factors associated with mental health problems in parents of children with autism. Our findings showed that symptoms of depression, anxiety and stress are common among parent caregivers of children with ASD. Perceived lack of social acceptance was the most significant factor contributing to psychological distress of parents, while friends' social support and employment status were found to be protective factors for parental mental health wellbeing in families caring for children with autism.

Clinically significant depressive symptoms were reported by 37.5% of caregivers of autistic children in our study, which was similar to other findings. A recent meta-analysis by Schnabel *et al*. (2020) identified overall prevalence of depressive disorders of 31% among parents of children with ASD, though other individual studies have shown variability in these findings. These disparate findings may be due to differences in the scales utilized, what the scale was measuring, and/or real differences in the socio-cultural contexts of study populations. A large-scale study from the USA conducted by Cohrs and Leslie in 2017, utilizing medical health claims of more than 85000 families, found a much lower prevalence of depression of 12.5%, in contrast to the 37.5% in our study.

We found that parents with moderately-to-extremely severe levels of anxiety symptoms accounted for 47.8% of all respondents, and a total of 38.2% had symptoms of stress. While our findings are similar to the findings of the study from Oman by Al-Farsi *et al*. (2016) for anxiety (45.9%), there is some difference from our study in their prevalence measure of stress symptoms, with 46%. The Davis and Carter study (2008) conducted in the US utilizing the Beck Anxiety Inventory found a prevalence of 12% among parents having anxiety symptoms, contrasting with our 47.8% using the DASS-21 instrument. The prevalence of anxiety in our study population is also higher than the 33% found in a meta-analysis consisting of papers with self-reported measures (Schnabel *et al*., 2020).

Employment status of participants in our sample, mostly comprised of mothers, was associated with reduced levels of stress, similar to findings of a study from Australia by Einam and Cuskelly (2002), which showed that the number of hours of paid employment was inversely associated with reported psychological problems in mothers of young adults with multiple disabilities. Studies have found that maternal employment positively impacts mood, feelings of accomplishment, and a positive sense of identity (McCabe, 2010). Our findings provide further evidence that mothers' employment may be protective for mothers of children with developmental disabilities by increasing their social network, as suggested by previous findings (Einam and Cuskelly, 2002).

Our findings showed that parents of children with autism and other impairments who were participating in ABA therapy were no longer at increased risks for stress and mood disorders after controlling for the effects of parent's employment status and level of perceived social support from friends and society; these factors confounding the risks. Our findings were consistent with those of the study by Hastings and Johnson (2001), which found that parents involved in intensive home-based behavioral intervention for their young child with autism reported no difference in stress levels from that of other parents of children with autism. Only a few studies have considered the impact of ABA intensity on parents. Contrary to our findings, previous studies by Schwichtenberg and Poehlmann (2007) and Eikeseth *et al*. (2015) showed that mothers of children participating in ABA intervention reported elevated depressive symptoms, and those mothers who were more involved in their child's ABA program reported more personal strain. The intense requisites that ABA programs place on children and families were hypothesized to explain these findings. Further research is needed to address these inconsistencies.

A substantial number of previous studies showed protective effects of informal social support for parents' mental well-being in families of children with disabilities (Weiss, 2002; Zaidman-Zait *et al*., 2017; Picardi *et al*., 2018). Our findings additionally showed that among different types of informal support, friends' support was more effective in helping parents of autistic children cope with stress and psychological disorders, similar to findings of previous studies (Ekas *et al*., 2010; Drogomyretska *et al*., 2020). Informal social network applications, mostly WhatsApp, were reported as the main source of social, informational and emotional support for parents of children with ASD in Kazakhstan in a previous study by An *et al*. (2018). There is a strong sense of camaraderie and solidarity among these parents, and many of them have become close friends. The strong stress-buffering effect of friends' social support for parents of children with ASD found in our study might be channeled in part through these parental informal social networks.

Study findings showed that parents who reported social acceptance as unmet-or-extremely-unmet needs had significantly higher levels of stress, anxiety, and depression symptoms, as compared to those who reported social acceptance as adequately met. Our findings are consistent with those of a systematic review by Papadopoulos *et al*. (2019), which found that autism-related stigma, including public stigma and internalized stigma, negatively impact informal caregivers' mental health, including depression, anxiety, and psychological distress. Findings of previous studies suggest that the adverse impact of autism stigma upon caregiver mental health generally declines over time because parents form new trusted friends who accept their child's disability (Gray, 2002; Divan *et al*., 2012; Papadopoulos *et al*., 2019). Positive meaning of caregiving, self-esteem, social support and caregiver burden acted as a buffer against the negative influence of stigma on caregiver well-being (Werner and Shulman, 2013). Families with children with ASD in Kazakhstan reported experiences of social rejection and stigma linked to autism in their community, in the mainstream health care, and in the educational systems (An *et al*., 2018). Interventions are needed to increase parental social support networks that can buffer the adverse impacts of stigma on caregivers' mental health. Awareness-raising activities that target the general public and parents themselves are vital to combating stigma and improving psychological wellbeing of parents and the entire family of children with ASD.

### Strengths and limitations

The major strength of this study is that it examined a wide range of risk and protective factors associated with stress, anxiety, and depressive symptoms in parents of children with ASD in the context of a region of the world with limited research in this field. Several limitations should be considered when reviewing this study. Firstly, we utilized a cross-sectional study design which prevents us from drawing inferences about causality. Secondly, choosing a self-reported research instrument may be somewhat less valid that a clinical diagnosis. However, the internationally-validated widely-used standardized DASS that we utilized has been validated using clinical diagnosis as the gold standard and provides a comprehensive overview of the psychological burden among caregivers of children with ASD, including an assessment of severity of symptoms. Third, we did not control for the effect of severity of the autistic child's symptoms; however, in previous studies, indicators of parental cognition and social support were found to be more important predictors of parental mental health than child-centric variables (Falk *et al*., 2014). Fourth, a relatively small sample size could decrease the power of the study to reveal significant differences between groups. Finally, since the majority of participants were from urban areas, the generalizability of findings to a rural population is limited.

We recommend further studies to characterizing the psychological burden of parents by gender to inform the planning of more effective targeted interventions for improving the mental health of parents caring for autistic children. A longitudinal study design would help in understanding how the associations of social support acceptance with parental mental health changes over time, and whether social support provides short-term and/or long-term benefits. In addition, there is a sparsity of studies on the associations of rural *v.* urban residence and other culture-specific factors with autism stigma and the mental health of caregivers. We just conducted our study prior to the COVID-19 pandemic. Follow-up studies are recommended to examine how social isolation and further lack of services due to the pandemic has impacted the already distressed families caring for children with ASD. This may provide compelling evidence for planning future interventions aimed to improve the mental wellbeing and health outcomes of these families.

## Conclusion

This study highlights the concern that caregivers of children with autism predominantly from urban households are observed to be more vulnerable to stress, anxiety, and depression symptoms. Given the evidence that parental stress has an impact on the effectiveness of interventions for children with autism, a more holistic parent-centric approach is needed, which includes further psychological support for parents to reach favorable outcomes for interventions providing parenting-skills training. Significant findings in terms of risk factors associated with parental mood disorders, such as the lack of social acceptance and stigmatization highlighted the vital importance of awareness raising activities in the context of post-Soviet countries with emerging and fragmentary care provisions for children with ASD and their families. Increased attention needs to be given to the employment opportunities of parents of children with ASD and development of social support networks that can play a protective role for caregivers' mental health and well-being of the entire family.
